# Food Attentional Bias and Eating Disordered symptomology: The moderating role of cognitive reappraisal

**DOI:** 10.1192/j.eurpsy.2022.266

**Published:** 2022-09-01

**Authors:** L. Lev-Ari, H. Kreiner, O. Avni

**Affiliations:** 1 Ruppin Academic Center, Clinical Psychology, Emek Hefer, Israel; 2 Ruppin Academic Center, Behavior Sciences, Emek Hefer, Israel; 3 Ruppin Academic Center, Gerontological Clinical Psychology, Emek Hefer, Israel

**Keywords:** food attention bias, eating disorder symptomology, cognitive reappraisal

## Abstract

**Introduction:**

Cognitive reappraisal (CR) is a cognitive regulation strategy aimed at changing how people think about certain stimuli in order to change their emotional impact. CR strategies have been used in research to try to change eating behaviors and other food-related responses. This study is the first to use a behavioral measurement to examine the effect of CR on food attentional bias (FAB) in people with elevated FAB.

**Objectives:**

It was hypothesized that CR would reduce FAB. Ninety-five participants were randomly assigned to one of three groups: CR, upregulation (UP) or controls (CN).

**Methods:**

All participants performed a computerized Visual Dot Probe (VDP) task using food stimuli to measure their FAB before and after the manipulation. The CR group recited five sentences aimed at curtailing the reward of high caloric food. Participants in the UP group recited five sentences aimed at strengthening the reward of high caloric food. The CN group recited five mundane sentences about their day. Participants also self-reported on eating disordered symptomology and BMI.

**Results:**

People with elevated FAB had more disordered eating than people low on FAB. A significant interaction was observed between group and time (pre/post-test), with the lowest FAB levels in the CR group following the manipulation.

**Conclusions:**

CR, a self-administered strategy can be effective in reducing FAB. CR may be an effective strategy for developing resistance to tempting food stimuli and curbing high caloric food intake. Being highly attentive to food cues may contribute to obesity. The attentional bias paradigm can be used to detect early signs of FAB.

**Disclosure:**

No significant relationships.

